# Adverse childhood events and mental health problems in cancer survivors: a systematic review

**DOI:** 10.1007/s00520-023-08280-7

**Published:** 2024-01-04

**Authors:** Chris Hinnen, Emma von Haeseler, Frederiek Tijssens, Floortje Mols

**Affiliations:** 1 Department of Psycho-Oncology, LUMC Oncology Center, Leiden, the Netherlands; 2 Department of Medical Psychology, Spaarne Gasthuis, Haarlem, the Netherlands; 3 Department of Medical and Clinical Psychology, CoRPS - Center of Research On Psychological Disorders and Somatic Diseases, Tilburg University, Tilburg, the Netherlands; 4 Netherlands Comprehensive Cancer Organisation (IKNL), Utrecht, the Netherlands

**Keywords:** Adverse childhood events, Cancer, Distress, Depression, Anxiety, Fatigue, Trauma-informed care

## Abstract

**Purpose:**

The purpose of this study was to systematically review the literature on the association between adverse childhood events (ACEs) and mental health problems in cancer survivors.

**Methods:**

This review was conducted in line with PRISMA (Preferred Reporting Items for Systematic Reviews and Meta-Analyses) guidelines. Four databases (PubMed, PsychINFO, Web of Science, and Cochrane) were searched on 27–08-2023.

**Results:**

Of the 1413 references yielded by the literature search, 25 papers met inclusion criteria and were reviewed. Most studies were performed in the USA, most included breast cancer survivors, and the number of included participants ranged between 20 and 1343. ACEs were relatively prevalent, with self-report rates ranging between 40 and 95%. Having been exposed to ACEs was a risk factor for heightened levels of emotional distress, anxiety, depressive symptoms, and fatigue during cancer treatment. Results varied depending on the variables included, and per subscale, but were consistent across different cultures and heterogenous patient groups.

**Conclusion:**

The association between ACE and mental health outcomes was significant in most studies. In order to improve treatment for this vulnerable population, it may be necessary to screen for ACEs before cancer treatment and adjust treatment, for example, by means of trauma-informed care (TIC), which recognizes and responds to the impact of trauma on individuals seeking healthcare.

## Introduction

Childhood is a sensitive period in life, with rapid bodily, neurological, cognitive, emotional, and social development. Experiencing multiple adverse events during childhood such as losing a parent, physical abuse, or having a parent with a mental illness are known risk factors for physical and mental health problems in adulthood [1]. Adversity in early childhood might lead to lifelong impairments in health [2, 3]. People who experience more adverse events during childhood are more likely to develop chronic illnesses such as heart disease, respiratory disease, and cancer [4]. Also, they are susceptible for developing depression, anxiety, and posttraumatic stress disorders [5–7]. The reasons why adverse childhood events may lead to poor health are diverse and still not completely understood, but sustained activation of the stress response system is assumed to be at the heart of this relationship. That is, chronic negative environmental factors may lead to disruption of the neuroendocrine and immune systems, in brain development as well as in learning abilities and responses to stress in the future [8, 9]. These disruptions are in turn linked to poorer health outcomes and increased mental problems. Moreover, both attachment theory [10] and schema-based cognitive models of mental health problems [11] argue that people may develop maladaptive schematic representations of the self (e.g., as incompetent), others (e.g., as not to be trusted) and the world (e.g., as unsafe) when confronted with adversities during childhood. These schemas impact how people appraise and deal with relationships and stressors in life making them more prone to develop mental health problems. Adverse childhood events (ACE) may become especially deleterious when confronted with catastrophic events, such as a cancer diagnosis, later in life [12] as it activates the stress system and (maladaptive) schemas. Consequently, people with ACE may be susceptible to stress-related problems and may have more problems dealing with the adversities and challenges imposed by the illness, making them susceptible for mental health problems.

A large body of literature shows that cancer diagnosis and treatment may be associated with emotional problems, impaired quality of life, and chronic fatigue in a substantial subgroup of cancer survivors [13, 14]. Identifying people who are susceptible for developing mental health problems when confronted with cancer is essential as it may guide patient management and interventions. Whether people with ACE may be at risk of mental health problems when confronted with cancer is, however, less known.

Therefore, the aim of this study is to systematically review the literature on the association between ACE and mental health problems in cancer survivors. Insight into the relationship between ACE and mental health problems among cancer survivors may help to identify who is at risk for mental health problems. This knowledge might lead to the improvement of care for cancer survivors.

## Method

### Data sources and search strategy

A systematic review of the literature up to August 27th, 2023 was conducted in line with PRISMA (Preferred Reporting Items for Systematic Reviews and Meta-Analyses) guidelines. A total of four databases (PubMed, PsychINFO, Web of Science, and Cochrane) were searched for relevant papers. A combination of search terms from the following concepts was used: adverse childhood events AND cancer AND psychological outcomes. The detailed list of search terms associated with each concept included in the search is provided in Table 1. The search strategy and selection of papers were guided by the research question: “What is the association between ACEs and mental health problems in cancer survivors?” We used the definition of the American Cancer Society when using the term “cancer survivor,” meaning that we considered anyone who has ever been diagnosed with cancer, no matter where they are in the course of their disease, to be a survivor. When performing the search, a filter for language was applied, including only articles in the English language.

**Table 1:** Search terms used in the different databases to select original research on the association between ACEs and psychological outcomes

Adverse Childhood Events	((Adverse Childhood Experience*) OR (childhood adversit*) OR (childhood trauma*) OR (traumatic child*) OR (early life stress*) OR (adolescent trauma*) OR ( Childhood sorrow*) OR (childhood misfortune*) OR (problematic childhood) OR (traumatic childhood))
Cancer	((Neoplas*) OR (Tumor*) OR (Tumour*) OR (Cancer*) OR (malignan*) OR (oncolog*) OR (carcinoma*))
Psychological outcomes	((Outcome*) OR (Survival) OR (Mortality) OR ( treatment effect*) OR (Prognos*) OR (Therapy adjust*) OR (treatment adjust*) OR (therapy adapt*) OR (treatment adapt*) OR (Psychopatholog*) OR (Adapt*) OR (Adjustment*) OR (Coping mechanism) OR (Anxiet*) OR (Anxious*) OR (Depress*) OR (Quality of Life) OR (QOL) OR (Life qualit*) OR (affecti*) OR (emotional disturbance*) OR (post traumatic stress*) OR (posttraumatic stress*) OR (post-traumatic stress*) OR (psychosocial) OR (psycho-social) OR (psychological) OR (psychologic) OR (Psycholog*) OR (mental state) OR (Fear*) OR (PTSD))

### Selection procedure

One author participated in the process of literature retrieval. Articles retrieved from the database searches were exported to a reference library (EndNote) and combined into one database, in which duplicates were deleted. Then, two authors screened all articles based on title and abstract and excluded papers on irrelevant topics. After, the full texts of the articles not having been excluded were read and labeled by three authors to come to the final selection. Inconsistencies between authors during the review process were resolved through discussion until consensus was achieved.

Both observational (cross-sectional, cohort, retrospective, and longitudinal) and intervention studies were included. Articles were included if the study reported an ACE as a measure correlated to psychological outcomes. Articles with adult life stress instead of ACEs were excluded. Articles assessing the relationship of ACEs as a risk factor for cancer or as a correlate to screening behavior were also excluded. Furthermore, articles were excluded if the described study was not original research (e.g., a review article or letter to the editor), not peer-reviewed (e.g., conference proceeding, thesis), if the study population did not (only) exist of cancer patients, or if the study population consisted of children instead of adults.

### Data extraction

**Fig. 1:**
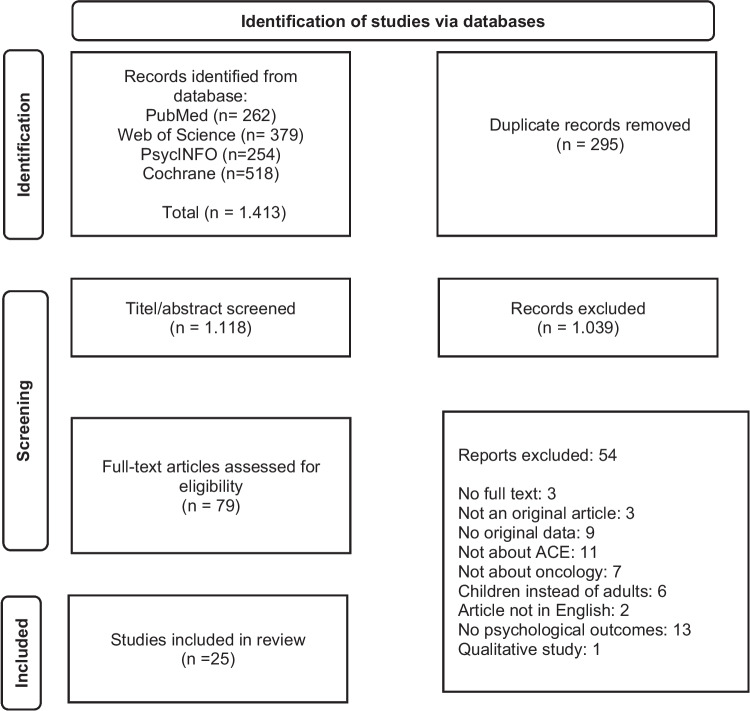
Flow-chart of inclusion and exclusion of publications derived from the database searches

For an overview of the number of papers in and excluded (see Fig. 1). For each article included in the present review, the following data were extracted and described: first author and year of publication, cancer population, sample size (including mean age), study design, ACE measurement used, prevalence of the ACEs.

## Results

In Fig. 1, a flow chart is depicted of the inclusion and exclusion of articles derived from the database searches. In total, 1413 references were found, and after the removal of 295 duplicates, 1118 unique articles were retrieved. These articles were assessed for eligibility and 79 full-text articles were assessed. Finally, 25 articles were included.

### General characteristics of the included studies

The majority of the 25 studies were conducted in the USA (*n* = 19, 76.0%) (see Table 2) [15–33]. The other studies were conducted in the UK [34, 35], Brazil [36], Turkey [37], and China [38, 39]. The number of participants in these studies ranged from 20 [21] to 1343 [31]. Median sample size was 110 (Q1 = 64, Q3 = 271, IQR = 207). Most of the studies were conducted in breast cancer survivors (*n* = 16, 64%) [16–25, 29, 30, 33, 35, 37, 38]. One study was conducted in lung cancer patients [27], one in head and neck squamous cell cancer (HNSCC) patients [36], one in ovarian cancer patients [15], and one in hematologic cancer survivors [26]. Five studies (20.8%) were conducted in survivors of mixed cancer types [28, 31, 32, 34, 39].

**Table 2:** Overview of the included studies

Reference	Country	Cancer type	N	Age, mean (SD)	Questionnaire	Prevalence of ACE
Archer (2012) [34]	UK	CRC and HNC	90	70.0 (10.0)	CTQ	Patients with CRC: *M* = 34.45 (SD = 7.49) Patients with HNC: *M* = 42.74 (SD = 17.53)
Armer (2018) [15]	USA	Ovarian cancer	337	59.7 (11.7)	LEDS & CTES	LEDS: adversity present in *N* = 59 (46.5%) CTES: *M* = 0.97 (SD = 1.06)
Banou (2009) [32]	USA	Heterogenous	64	53.4 (11.3)	CTQ (7-items)	38 patients experienced at least one incidence of childhood adversities (59.4%) CTQ: *M* = 2.56 (SD = 4.17)
Bower (2014) [16]	USA	Breast cancer	50	57.8 (unknown)	CTQ	Fatigued patients *M* = 2.45 (SD = 1.1) and non-fatigued patients *M* = 1.56 (SD = 0.6)
Bower (2018) [17]	USA	Breast cancer	191	51.8 (8.3)	RFQ	*M* = 28.06 (SD = 10.50)
Bower (2019) [18]	USA	Breast Cancer	270	56.0 (11.0)	CTQ	40% were exposed to some form of childhood maltreatment. 28% of the total sample (*N* = 75) of those reported non-sexual maltreatment and 12% (*N* = 32) reported sexual maltreatment
Fagundes (2012) [19]	USA	Breast cancer	132	51.7 (9.5)	CTQ	48% were exposed to some form of childhood maltreatment
Goldsmith (2010) [20]	USA	Breast cancer	330	50.7 (9.9)	CTQ	Emotional abuse: *M* = 7.85 (SD = 4.04) Physical abuse: *M* = 6.65 (SD = 2.94) Sexual abuse: *M* = 6.34 (SD = 3.16)
Guveli (2017) [37]	Turkey	Breast cancer	310	51,5 (11.4)	CTQ	54.4% of participants experienced at least one type of childhood maltreatment
Han (2016) [21]	USA	Breast cancer	20	57 (11.6) No-ACE 47.4 (6.5) ACE	CTQ	40% of participants (*N* = 8) scored above the cutoff scores for the CTQ
Kamen (2017) [22]	USA	Breast cancer	56	53.6 (9.8)	TES	41.% (*N* = 23) had experienced one traumatic event, 10.7% (*N* = 6) had experienced two traumatic events, 3.6% (*N* = 2) had experienced three and one participant had experienced four traumatic events
Kanzawa-Lee (2020) [23]	USA	Breast cancer	44	56.5 (11.3)	CTES	50% (*N* = 21) had experienced traumatic events
Kuhlman (2017) [24]	USA	Breast cancer	271	56.2 (11.5)	CTQ	39.5% of participants had experienced childhood maltreatment CTQ: *M* = 36.04 (SD = 13.26)
McFarland (2016) [25]	USA	Breast cancer	125	55.4 (13.2)	RFQ	*M* = 23.31 (SD = 10.03)
McFarland (2017) [26]	USA	Hematologic cancer	117	57.7 (14.8)	RFQ	*M* = 25.04 (SD = 10.34)
McFarland (2020) [27]	USA	Lung cancer	92	65.4 (9.2)	RFQ	*M* = 23.9 (SD = 9.3)
Romanovska (2022) [31]	USA	Heterogenous	1343	Group 0 (*N* = 371) 58.2 (11.9) Group 1 (*N* = 519) 58.5 (12.5) Group 2 (*N* = 453) (54.8 (12.3)	LSC-R	Group 0 (no pain and moderate sleep disturbance) *M* = 4.8 (SD = 3.2), Group 1 (moderate pain and moderate sleep disturbance) *M* = 5.8 (SD = 3.6), Group 2 (severe pain and high sleep disturbance) *M* = 7.3 (SD = 4.5) Family violence in childhood: 19 (*N* = 49) of patients in Group 0, 21.6% (*N* = 91) in Group 1, and 29.9% (*N* = 103) in Group 2 Physical abuse < 16: 10% (*N* = 26) of Group 0, 13.3% (*N* = 49) of Group 1 and 18.8% (*N* = 65) of Group 2 Forced to touch < 16: 6.2% (*N* = 16) of Group 0, 9.1% (*N* = 38) of Group 1 and 18.6% (*N* = 65) in Group 2 Forced to have sex < 16: 1.6% (*N* = 4) in Group 0, 2.9% (*N* = 12) in Group 1 and 8.3% (*N* = 29) in Group 2
Roh (2019) [28]	USA	Heterogenous	73	56.5 (8.4)	ACEQ	*M* = 2.5 (SD = 2.3)
Salmon (2007) [35]	UK	Breast cancer	355	58.2 (10.2)	Self-report questions on sexual, physical, and emotional abuse	41% of participants (*N* = 141) recalled at least one experience of abuse. 10% reported sexual abuse, 28% physical, and 25% reported emotional abuse
Sarafim-Silva (2018) [36]	Brazil	Head and Neck Cancer	110	0–45 (*N* = 9, 8.1%) 46–65 (*N* = 72, 65.5%) > 65 (*N* = 29, 26.4%)	CTQ	95.5% (*N* = 105) of participants experienced at least one type of ACE
Tell (2018) [29]	USA	Breast cancer	29	52.3 (10.4)	CTQ	*M* = 36.14 (SD = 17.84)
Janusek (2013) [40]	USA	Breast cancer	40	55.6 (9.4)	CTQ	Physical neglect: *M* = 6.6 (SD = 2.6) Emotional neglect: *M* = 9.2 (SD = 4.9) Emotional abuse: *M* = 8.9 (SD = 4.9) Physical abuse: *M* = 6.9 (SD = 4.3) Sexual abuse: *M* = 6.8 (SD = 3.4)
Janusek (2021) [33]	USA	Breast cancer	84	55.2 (9.7)	CTQ	29 participants completed the CTQ. Data are not specified
Xue (2016) [38]	China	Breast cancer	93	51.76 (8.9)	ACEQ	61 participants (65.5%) experienced no ACE, 15 experienced one kind of ACE (16.1%) and 17 participants experienced more than two kinds of ACE (18.3%)
Zhang (2020) [39]	China	Heterogenous	603	47.7 (11.5)	Non-validated questions	30 participants (5.0%) experienced at least one ACE

### Type of ACE measurements

The Childhood Trauma Questionnaire (CTQ) (or subscale) was used to measure ACE in more than half of the studies (*n* = 13, 52%) [16, 18–21, 24, 29, 32–34, 36, 37, 40]. The CTQ is a 28-item inventory that provides a reliable and valid screening for a history of abuse and neglect [41]. Four studies [17, 25–27] used the Risky Families Questionnaire (RFQ)[42], one study [22] used the Traumatic Events Survey (TES)[43], two studies [15, 23] used the Childhood Traumatic Events Scale (CTES) [44], one study [38] used the Adverse Childhood Experience Questionnaire (ACEQ)[45], one; [28] the ACE Questionnaire by the Center of Disease Control (CDC)[46], and one [31] the Life Stressor Checklist-Revised (LSC-R)[47]. Furthermore, the semi-structured interview Life events and difficulties schedule (LEDS) [48] was used [15], and one study used non-validated self-report questions regarding ACEs [35]. The percentage of patients who reported at least one incidence of ACE ranged from approximately 40.0 [18] to 95.5% [36]. For a detailed overview of the studies and the prevalence of ACE, see Table 2.

### ACEs and mental health

#### Depression and anxiety

The association between exposure to ACEs and depression was investigated in 12 of the 24 included studies [21, 22, 24–28, 30, 32, 34, 36, 37]. Childhood adversities were significantly associated with higher levels of depressive symptoms in patients with cancer in 10 of these studies [21, 24–27, 30, 32, 34, 36, 37]. In two studies [22, 28] no association between ACE and depression was found.

Nine studies investigated the relationship between ACEs and anxiety [15, 22, 23, 25–27, 29, 36, 37]. Associations with higher levels of anxiety were found in seven of these studies [15, 25–27, 29, 36, 37]. In two studies, elevated scores of depression and anxiety were associated with ACEs in univariate analyses, while in multivariate analysis involving depression, anxiety, distress, and/or physical symptoms only the relationship between ACEs and depression remained significant [25, 27].

Some studies looked not only at childhood adversities in general (i.e., total score) but also at specific adversities (i.e., subscales) [25, 26, 30, 36]. In one study all the subscales of the RFQ were significantly associated with higher levels of anxiety, depression, and distress [25], while in another study using the RFQ, differences between the types of adverse events were found [26]. That is, the abuse subscale was associated with distress, the chaotic home environment was associated with higher levels of distress and anxiety, and the neglect subscale was not associated with any of these outcomes [26]. Additionally, the CTQ subscales were found to be differently associated with psychological variables. That is, physical neglect was found to be associated with higher anxiety levels, whereas physical abuse and emotional neglect were not. Emotional abuse, physical abuse, and physical neglect were all associated with higher levels of depression [36]. In a study among breast cancer survivors using the CTQ, emotional neglect and abuse were associated with higher initial levels of depression, but not with changes in depressive symptoms over time, whereas physical neglect was a significant predictor of higher levels of stress over time, but not of the initial stress level [40].

#### Fatigue

Seven studies investigated the relationship between ACEs and fatigue during and/or after cancer treatment. Cancer patients who had been exposed to ACEs experienced higher levels of fatigue in six of the seven studies [16–19, 21, 40]. One study identified five distinct groups of fatigue trajectories: women who experienced consistently low, low and decreasing, low and then increasing, high and then decreasing, and persistently elevated levels of fatigue. Women who had been exposed to ACEs more were more likely to suffer from consistently high levels of fatigue or experienced higher levels immediately after treatment and then recovered rather than having low and then increasing levels of fatigue [17]. In a study among breast cancer survivors [16], a dichotomous ACE score was associated with higher fatigue, but the severity of ACE was not. In another study among breast cancer survivors, the emotional neglect and abuse subscales of the CTQ were associated with initial fatigue levels, but not with changes in fatigue over time [40]. Moreover, survivors who suffered from severe pain and high sleep disturbance reported the highest rates for family violence in childhood, forced touching, and forced sex at an age younger than 16 compared to people who did not suffer from pain and reported moderate sleep disturbance, or moderate pain and moderate sleep disturbance [31]. Regarding physical abuse, the difference was only significant between the severe and the no pain group [31].

#### Other psychological variables and mechanisms

Moreover, ACEs were also found to be associated with more cancer-related traumatic symptoms [32]. Specifically, intrusive thoughts were correlated with having experienced emotional, physical, and sexual abuse [20]. Furthermore, exposure to ACEs was associated with elevated levels of cancer-related psychological distress [19, 26, 27], perceived stress [21, 30], sleep disturbance and sleep-related impairment [23], and suicidal ideation [39]. Moreover, ACEs were also associated with worse emotional well-being [19], worse quality of life [30, 38], and with an increase in negative adjustment and a decrease in positive adjustment after cancer [37].

Other influential relationships were investigated. Social support seems to mediate the relationship between ACEs and quality of life [19] and marital status may buffer the effect of childhood adversities on fatigue and depression [21]. Moreover, women with breast cancer who experienced ACE showed an elevated cortisol and proinflammatory cytokine release, especially when showing reduced parasympathetic activity during real-time stress (Trier Social Stress Test) [29]. It was also found that the differences in psychological well-being between people who had been exposed to ACEs compared to those who had not were similar during the diagnostic and the treatment phase [21]. Additionally, one study found that people with ACEs experienced less social and professional support during cancer treatment [35] and another study [33] showed that they may profit from mindfulness-based therapy.

## Discussion

The aim of this systematic literature review was to investigate the association between ACEs and psychological problems in cancer survivors. Although variations were found, and not all studies reported an association between ACEs and mental health problems in cancer survivors, the majority did. On the basis of this review, it seems safe to state that ACEs are prevalent (> 50%) and seem to be a risk factor for more emotional distress, anxiety, depressive symptoms, and fatigue in cancer survivors. If this is true, the next question is what to do with this knowledge?

The most obvious option is to start screening patients on whether or not they have experienced ACEs. This may, however, be rather disturbing for patients [49], and physicians and nurses might be reluctant to do this with questionnaires as it is experienced as too upsetting for patients [50]. A more viable option might be to teach the medical staff to ask patients whether or not they have had experiences during childhood, that they feel may impact their needs and abilities during the illness process [8]. In a study by Clark (2014), women with breast cancer emphasized the importance of asking about adversities (including abuse) as it may give them the opportunity and choice to disclose adversities and thereby tailor support [49]. Recently, scholars have started to develop ideas about how health care can be adapted in such a way that it takes childhood adversities into account [9, 51]. This so-called trauma-informed care (TIC) recognizes and responds to the impact of trauma on individuals seeking healthcare. It is an approach that emphasizes safety, trustworthiness, choice, collaboration, and empowerment for individuals who have experienced trauma [52].

While this review suggests that ACE is a risk factor for psychological problems in cancer survivors, many questions remain unanswered. First, are specific ACEs a risk factor for specific psychological problems (e.g., anxiety, depression, PTSD) in patients with cancer? Previous research, among non-somatically ill patients, suggests that different types of adversities (e.g., neglect, abuse) make people susceptible for specific mental health problems (anxiety, depression, PTSD) [53, 54]. For example, Veen et al. (2013) [55] found that emotional neglect was particularly associated with anhedonia and sexual abuse with anxious arousal.

Moreover, do specific childhood adversities have a specific impact on patients with cancer depending on illness characteristics such as illness phase (i.e., diagnostic, treatment, follow-up, palliative) and type of treatment? It could be argued that certain ACEs may be especially influential during the treatment process. For example, for patients who have experienced sexual trauma, brachytherapy, where a radiation source is placed inside the vagina or prostate, may be particularly distressing and potentially retraumatizing [56]. Other ACEs such as emotional abuse may for example have an especially deleterious effect on emotional recovery after treatment.

Furthermore, not only the type of adversity may have a specific impact on mental health, but also the frequency since previous studies suggested that ACEs have a dose–response effect on health [42] with more ACEs (> 2) being more deleterious than one or two. Whether this is also true for people’s mental health when confronted with cancer is unclear. Moreover, the majority of included studies were conducted in Western countries and in women with breast cancer and therefore may not be generalizable to cancer patients in general.

Another topic in which many questions remain largely unanswered is the mechanisms by which ACEs influence mental health problems in cancer survivors. In general, ACEs are believed to influence (mental) health through biological (altered stress and inflammatory responses, epigenetic alterations), psychological (developmental maladaptive schemas, cognitive distortions, unhealthy behaviors), and social (adverse social circumstances) processes [57–60]. How these mechanisms play a role in the different mental health problems survivors face (i.e., fatigue, anxiety, anhedonia, separation distress) may be a focus of attention in future studies.

## Conclusion

Childhood adversities are prevalent and a risk factor for psychological problems in patients diagnosed with cancer. Recognizing the prevalence of ACE and its impact on mental health in cancer survivors and responding in a way that prevents re-traumatization and promotes resilience should become a focus of attention in cancer care.
